# Culturally Responsive Counseling and Tuberculosis Treatment Adherence: Development and Validation of an Integrated Model

**DOI:** 10.3390/nursrep16060190

**Published:** 2026-05-29

**Authors:** Era Dorihi Kale, Nursalam Nursalam, Makhfudli Makhfudli, Rapin Polsook, I Gede Juanamasta

**Affiliations:** 1 Faculty of Nursing, Airlangga University, Surabaya 60115, Indonesia; nursalam@fkp.unair.ac.id (N.N.); makhfudli@fkp.unair.ac.id (M.M.); 2 Ners Program, Poltekkes Kemenkes Kupang, Kupang 85111, Indonesia; 3 Faculty of Nursing, Chulalongkorn University, Bangkok 10330, Thailand; rapin.p@chula.ac.th (R.P.);

**Keywords:** tuberculosis, treatment adherence, transcultural nursing, Sunrise Model, IMB framework, culturally grounded counseling, SEM-PLS, Indonesia, cross-sectional study

## Abstract

**Background/Objectives**: Tuberculosis (TB) treatment adherence remains suboptimal globally, contributing to relapse, transmission, and drug-resistant TB. Adherence is a culturally embedded behavioral process shaped by psychological readiness, social relationships, and structural conditions. This study aimed to validate a culturally grounded counseling model integrating the Sunrise Model of transcultural nursing and the Information–Motivation–Behavioral Skills (IMB) framework for TB treatment adherence in Kupang City, Indonesia. **Methods**: A cross-sectional study enrolled 172 pulmonary TB patients across all 11 primary healthcare centers in Kupang City (June–September 2025). A validated questionnaire measuring eight transcultural determinants, culturally grounded counseling (mediator), and adherence (outcome) was developed through expert validation, cognitive interviewing, and pilot testing. Structural Equation Modeling–Partial Least Squares (SEM-PLS) tested structural and mediation relationships via bootstrapped indirect effects (*p* < 0.05). **Results**: Reliability (CR: 0.842–0.959; ρA: 0.791–0.957), convergent validity (AVE: 0.577–0.921), and discriminant validity (all HTMT < 0.85) were confirmed. The model showed strong explanatory power (R^2^ = 0.649 for adherence; SRMR = 0.074). Culturally grounded counseling was positively associated with adherence (β = 0.245, *p* = 0.003) and statistically mediated five antecedent–adherence relationships, including full mediation for economic conditions. Cultural values and lifestyle showed the strongest counseling association (β = 0.345, *p* < 0.001). **Conclusions**: Cross-sectional evidence supports a culturally grounded counseling model for TB adherence in diverse settings. Causal conclusions require longitudinal and interventional validation. The model offers a foundation for nurse-led intervention development and multi-site validation.

## 1. Introduction

Tuberculosis (TB) remains one of the foremost causes of morbidity and mortality from a single infectious agent worldwide. The World Health Organization (WHO) estimated 10.8 million new TB cases and 1.25 million TB-related deaths globally in 2023, with the highest burdens concentrated in South and South-East Asia and sub-Saharan Africa [1]. Despite the existence of effective six-month first-line pharmacological regimens [2], global TB control continues to be undermined by suboptimal treatment adherence, which remains one of the principal drivers of poor treatment outcomes and the emergence of drug-resistant disease [3,4]. Indonesia carries the second-highest TB burden globally, with an estimated 1,060,000 new cases and 134,000 deaths in 2023 [1,5].

Historically, TB control programs have addressed adherence through standardized structural interventions, most notably directly observed therapy (DOT) and patient education campaigns. Although these strategies have produced measurable improvements in some settings, evidence consistently indicates that adherence remains suboptimal across diverse populations [1]. A systematic review of qualitative TB adherence research identified a complex array of patient-level, social, and structural factors that DOT and education alone cannot adequately address, including stigma, household poverty, competing livelihood demands, and inconsistent side effect management [6]. These findings suggest that adherence behavior cannot be fully explained or addressed through biomedical frameworks or medication access alone.

From nursing and behavioral science perspectives, adherence is increasingly conceptualized as a multidimensional behavioral process embedded in patients’ psychological states, social environments, cultural belief systems, and structural conditions [3,6,7]. Transcultural nursing theory, specifically Leininger’s Culture Care Diversity and Universality Theory operationalized through the Sunrise Model, offers a comprehensive framework for understanding how cultural values and belief systems shape health behaviors and health-seeking practices [8,9]. The Sunrise Model delineates seven cultural and structural dimensions: kinship and social relationships; religious and philosophical beliefs; educational background; technological factors; economic conditions; political and legal context; and cultural values and lifestyle [8,9,10,11].

Complementing this cultural perspective, the Information–Motivation–Behavioral Skills (IMB) framework provides a theoretically grounded behavioral science account of treatment adherence [12,13,14]. The IMB model proposes that three proximal determinants predict health-related behavior: relevant information, motivation (personal and social), and behavioral skills (objective capacity and self-efficacy) [13]. The framework has been validated across multiple chronic disease contexts, including antiretroviral therapy for HIV and TB treatment [12,13].

The conceptual integration of these two frameworks is complementary and synergistic. The three IMB components can be mapped explicitly onto specific Sunrise Model dimensions: (1) Information maps onto educational factors and technological access, which shape patients’ knowledge about TB, its treatment rationale, and available support resources. (2) Motivation maps onto religious and philosophical beliefs, social and kinship relationships, and psychological determinants. (3) Behavioral skills map onto cultural values and lifestyle and economic conditions, which determine whether regimen routines are feasible within daily obligations and structural constraints. Culturally grounded counseling emerges from this integration as the clinical nursing process through which cultural dimensions are translated into the three IMB elements in a manner coherent with patients’ worldviews, social structures, and material realities. This theoretical mapping provided the conceptual basis for the study’s structural model.

The setting of this study, Kupang City, the capital of East Nusa Tenggara (NTT) Province, Indonesia, provides an important yet underrepresented context for TB adherence research. NTT is one of Indonesia’s most ethnically and linguistically diverse provinces, home predominantly to Timorese, Rotenese, Savunese, and Sumbanese communities, each with distinct customary (*adat*) traditions, kinship structures, and cosmological belief systems [15]. Religiously, NTT is predominantly Christian (Protestant and Catholic), contrasting with the majority-Muslim, Javanese-dominant cultural milieu characterizing most published Indonesian TB adherence research. NTT also reports among the highest TB notification rates in Indonesia, compounded by geographic remoteness, limited primary healthcare infrastructure, high rates of food insecurity, and elevated poverty relative to the national average [1].

Although prior studies have examined cultural determinants of TB treatment adherence [7,16,17] and evaluated counseling-based interventions [18], no study has integrated transcultural nursing theory and the IMB behavioral framework into a single, empirically tested structural model that positions culturally grounded counseling as a theorized mediating mechanism. Most existing research has examined cultural or behavioral determinants in isolation, and the majority of Indonesian TB adherence research has been conducted in Javanese or Balinese contexts. This study addresses that gap and makes three distinct contributions compared with prior work: (1) it integrates the Sunrise Model and IMB framework into a single structural model rather than applying them in parallel; (2) it empirically tests culturally grounded counseling as an explicit mediating mechanism linking cultural dimensions to adherence behavior; and (3) it validates the model in the Eastern Indonesian setting of NTT—a Christian-majority, multi-ethnic province that has been largely absent from the Indonesian TB adherence literature. Therefore, this study aimed to develop and validate a culturally grounded counseling model integrating the Sunrise Model and the IMB framework, using SEM-PLS to examine structural associations and the statistical mediating role of culturally grounded counseling in TB treatment adherence among patients in Kupang City, Indonesia.

## 2. Materials and Methods

### 2.1. Study Design

This study employed a cross-sectional design to develop and validate a theoretical model of TB treatment adherence. SEM-PLS was used to examine relationships among cultural, behavioral, and contextual determinants. SEM-PLS was selected for its suitability for theory development and complex models with multiple latent constructs [19]. The study is reported in accordance with the STROBE (Strengthening the Reporting of Observational Studies in Epidemiology) checklist for cross-sectional studies. Because the design is cross-sectional, all structural path estimates and mediation findings are interpreted as statistical associations consistent with the proposed theoretical model, not as evidence of causal relationships.

### 2.2. Setting

In Indonesia, TB management operates across three tiers: Puskesmas (primary healthcare centers), district hospitals, and provincial or referral hospitals. Newly diagnosed, drug-sensitive pulmonary TB is managed primarily at the Puskesmas level under the Indonesian National TB Elimination Program and WHO DOTS guidelines. The standard first-line regimen consists of an intensive phase of two months of isoniazid, rifampicin, pyrazinamide, and ethambutol (2HRZE), followed by a continuation phase of four months of isoniazid and rifampicin (4HR), totaling six months. The study was conducted across all 11 Puskesmas in Kupang City that provided TB treatment services from June to September 2025.

### 2.3. Participants and Sampling

The target population comprised all adult patients with pulmonary TB who were actively receiving first-line treatment at any of the 11 Puskesmas in Kupang City during June–September 2025. A total (census) sampling approach was applied—not convenience sampling—meaning that every eligible patient registered on the active TB treatment roster at each facility during the study period was invited to participate. No probabilistic sampling was required since the entire accessible population was enrolled. A total of 172 participants were enrolled (response rate: 100%).

Inclusion criteria: (1) confirmed diagnosis of pulmonary TB by sputum smear microscopy or GeneXpert MTB/RIF assay; (2) aged ≥18 years; (3) currently enrolled in first-line TB treatment at one of the 11 Puskesmas; and (4) able to provide written informed consent.

Exclusion criteria: (1) extrapulmonary TB only; (2) concurrent MDR/XDR-TB; (3) severe cognitive impairment; and (4) >20% of items unanswered.

Participants were classified into the intensive phase (months 1–2: 2HRZE) or continuation phase (months 3–6: 4HR) based on their treatment month as recorded in the individual TB treatment card (form TB-01), verified by the treating nurse or TB program officer.

### 2.4. Sample Size Justification

Sample size adequacy was evaluated using two criteria. Applying the 10-times rule [19], the minimum required sample size equals 10 times the largest number of structural paths directed at any single construct (maximum of eight paths directed at Y1), yielding a minimum of 80 participants, which our sample of 172 substantially exceeds. Using the inverse square root method [20], the minimum sample required to detect a path coefficient of 0.20 at α = 0.05, power = 0.80 is approximately 153 participants; our sample satisfies this criterion. The participant-to-indicator ratio (172:28 = 6.1:1) also exceeds the minimum of 5:1 recommended for reflective PLS models.

### 2.5. Instrument Development

A new structured questionnaire was developed because no existing instrument adequately captured the integration of all eight Sunrise Model dimensions, culturally grounded counseling as a distinct mediating construct, and TB treatment adherence within the NTT sociocultural context. Instrument development followed a systematic four-stage process: (1) Theoretical item generation through literature review and explicit mapping to each Sunrise Model dimension and IMB construct. (2) Expert content validation by a panel of five specialists—comprising (a) a transcultural nursing academic with expertise in Leininger’s Culture Care Theory, (b) a TB clinical nurse specialist with more than 10 years of clinical experience in NTT, (c) a public health researcher specializing in TB program evaluation in Eastern Indonesia, (d) a psychologist with expertise in health behavior measurement and adherence, and (e) a community health nurse from Kupang with direct Puskesmas-based TB care experience—who independently rated each item on content relevance, cultural appropriateness, clarity, and wording using a four-point scale. Items with item-level content validity index (I-CVI) below 0.80 were revised or removed; the overall scale-level CVI (S-CVI/Ave) was 0.91, exceeding the recommended 0.90 threshold [21]. (3) Cognitive interviewing with five TB patients not included in the main sample. (4) Pilot testing with 30 patients (item-total correlation > 0.30; preliminary factor loading > 0.40).

The final questionnaire comprised ten constructs measured by 28 retained indicators (after removal of two items during pilot analysis): psychological determinants (X1; 3 items; e.g., ‘I am confident I can complete my full course of TB treatment’); educational factors (X2; 3 retained items; e.g., ‘I understand why I must take my TB medication every day without missing a dose’); technological factors (X3; 2 items; e.g., ‘I use my mobile phone to set reminders to take my medication on time’); cultural values and lifestyle (X4; 2 items; e.g., ‘My cultural or community obligations sometimes make it difficult to maintain my daily medication schedule’); religious and philosophical beliefs (X5; 2 items; e.g., ‘My faith community encourages and supports me to continue TB treatment’); social and kinship relationships (X6; 2 items; e.g., ‘My family or clan members accompany me to clinic appointments and remind me to take my medication’); economic conditions (X7; 3 items; e.g., ‘The cost of transportation to the health center makes it difficult for me to attend all appointments’); political–legal context (X8; 2 retained items; e.g., ‘I am aware of my legal rights and protections as a TB patient’); culturally grounded counseling (X9; 4 items; e.g., ‘The nurse or health worker explained my treatment plan using language and examples that matched my cultural background and daily life’); and TB treatment adherence (Y1; 4 items assessing daily medication intake, clinic attendance, full-course completion, and side-effect management without treatment interruption; e.g., ‘I take every dose of my TB medication as prescribed, every day, without missing any’). All items were rated on a five-point Likert scale (1 = strongly disagree to 5 = strongly agree). The complete questionnaire, including all item wordings, response scale anchors, can be requested upon reasonable request.

### 2.6. Data Collection

Data were collected by trained research nurses at each Puskesmas following scheduled treatment appointments. Questionnaires were self-administered, with standardized interviewer assistance available. Data collection was conducted in private consultation rooms. To reduce social desirability bias, research nurses emphasized confidentiality and the independence of the study from clinical care. Nurses were trained to use pre-specified neutral probes only during assisted completion.

### 2.7. Data Analysis

Descriptive statistics were computed for all sociodemographic variables and construct scores (frequencies, percentages, means, and standard deviations) using IBM SPSS Statistics 26.0 (IBM Corp., Armonk, NY, USA). SEM-PLS was conducted using SmartPLS 4.0 (SmartPLS GmbH, Bönningstedt, Germany), following a two-stage procedure [19]. Measurement model evaluation examined outer loadings, composite reliability (CR), Dijkstra–Henseler’s rho_A (ρA) as a more conservative reliability estimate, average variance extracted (AVE), and discriminant validity using the heterotrait–monotrait ratio (HTMT). Common method bias (CMB) was assessed using Harman’s single-factor test (threshold: first-factor variance < 50%). Although Harman’s test has been critiqued as insufficiently sensitive when used in isolation [22], it was supplemented here by variance inflation factor (VIF) examination of all indicator pairs—VIF values below 3.3 provide additional evidence against dominant common method variance [19]. Prior to structural model estimation, three prerequisite assumptions were verified: (1) multicollinearity among predictor constructs was assessed using VIF values (acceptable threshold < 5.0); (2) independence of residuals was evaluated using the Durbin–Watson statistic (acceptable range: 1.5–2.5); and (3) homoscedasticity was assessed through visual inspection of residuals versus fitted value plots. Structural model evaluation then assessed path coefficients (β) via 5000-resample bootstrapping with bias-corrected 95% confidence intervals, R^2^ values for explanatory power, Stone–Geisser Q^2^ statistics via blindfolding (omission distance = 7) for predictive relevance, and f^2^ effect sizes (small ≥ 0.02, medium ≥ 0.15, large ≥ 0.35) [19]. Statistical mediation was examined via bootstrapped indirect effects (*p* < 0.05), classified as full (non-significant direct, significant indirect) or partial (both significant).

### 2.8. Ethical Considerations

This study was approved by the Health Research Ethics Committee of the Faculty of Nursing, Airlangga University (Approval No. 3387-KEPK), and conducted in accordance with the Declaration of Helsinki. Written informed consent was obtained from all participants. Participation was voluntary, with the right to withdraw at any time without consequences to treatment.

## 3. Results

### 3.1. Participant Characteristics

A total of 172 patients with pulmonary TB were enrolled across all 11 Puskesmas in Kupang City with no missing data (response rate: 100%). All percentages reported below refer to the total sample of n = 172. Table 1 presents participant sociodemographic, treatment, and construct score characteristics. The sample was predominantly composed of adults aged 19–44 years (n = 118; 68.6%), with a mean age of 35.7 years (SD = 13.4; 95% CI: 33.7–37.7), consistent with the productive-age TB epidemiological profile in Indonesia. Males constituted 54.7% (n = 94). The majority had completed senior high school (n = 98; 57.0%). Diverse occupational backgrounds were represented, including self-employed individuals (n = 40; 23.3%) and homemakers (n = 30; 17.4%), reflecting the informal economy structure of Kupang City. The majority of participants (n = 107; 62.2%) were in the continuation phase of treatment at data collection, consistent with the expected distribution across an active TB treatment cohort. Construct score descriptive statistics (all rated 1–5) are presented in Table 1. Mean scores ranged from 3.29 (SD = 0.84) for economic conditions (X7) to 3.94 (SD = 0.62) for religious and philosophical beliefs (X5), indicating moderate-to-high endorsement across all dimensions. The TB treatment adherence construct (Y1) had a mean score of 3.77 (SD = 0.66).

### 3.2. Measurement Model

Two indicators were removed during measurement model evaluation: X2.1 (loading = −0.077), which showed poor discriminant performance attributable to ceiling effects in a predominantly senior-high-school-educated sample, and X8.1 (loading = −0.317), which addressed trust in government health policy and was interpreted heterogeneously, yielding a non-informative negative loading. All remaining indicators and constructs met recommended psychometric thresholds (Table 2). CR values ranged from 0.842 to 0.959 (all > 0.70), and ρA values ranged from 0.791 to 0.957 (all > 0.70), confirming adequate reliability by both standard and conservative criteria. AVE values ranged from 0.577 to 0.921 (all > 0.50), confirming convergent validity. Maximum shared variance (MSV) was lower than AVE for all constructs, supporting discriminant validity. Harman’s single-factor test indicated that the largest unrotated factor accounted for 28.3% of total variance, well below the 50% threshold, suggesting CMB is unlikely to be a dominant confound. Two indicators with loadings below 0.70 (X2.2 = 0.575; Y1.4 = 0.551) were retained on grounds of theoretical irreplaceability and acceptable construct-level reliability and AVE. This result was supplemented by VIF examination of all indicator pairs (range: 1.55–2.44; all < 3.3), providing convergent evidence against dominant common method bias [22].

Table 3 presents the HTMT discriminant validity matrix. All coefficients ranged from 0.055 to 0.832, all below the 0.85 threshold [23], confirming acceptable discriminant validity. The highest value, X9–Y1 (HTMT = 0.832), reflects the theoretical proximity of counseling and adherence as proximal constructs in the model; X9 items measure the counseling process while Y1 items measure medication-taking and appointment attendance. The X6–X7 HTMT of 0.731 reflects the known empirical overlap between social relationships and economic conditions in resource-constrained settings.

### 3.3. Structural Model

The structural model demonstrated strong explanatory power: R^2^ = 0.658 for culturally grounded counseling and R^2^ = 0.649 for TB treatment adherence. Model fit was acceptable (SRMR = 0.074 < 0.08). Predictive relevance was confirmed: Q^2^ = 0.413 for counseling (moderate-to-substantial) and Q^2^ = 0.335 for adherence (moderate), following Hair et al.’s [19] thresholds. All predictor VIFs were below 3.3 (range: 1.55–2.44), confirming the absence of multicollinearity. The Durbin–Watson statistic was 1.97 (within the acceptable range of 1.5–2.5), confirming independence of residuals. Visual inspection of residuals versus fitted value plots confirmed homoscedasticity. All model assumptions were therefore satisfied prior to path interpretation.

Effect sizes (f^2^): Cultural values and lifestyle demonstrated a medium-to-large effect on counseling (f^2^ = 0.272), followed by psychological determinants (f^2^ = 0.160, medium). For direct paths to adherence, religious and philosophical beliefs showed the largest effect (f^2^ = 0.125, small-to-medium). The effect of culturally grounded counseling on adherence was small (f^2^ = 0.058), which is expected in a mediation model where indirect pathways accumulate. Full structural model results are presented in Table 4.

Psychological determinants (X1), cultural values and lifestyle (X4), religious and philosophical beliefs (X5), social and kinship relationships (X6), and economic conditions (X7) were each significantly associated with culturally grounded counseling. Educational (X2), technological (X3), and political–legal (X8) factors were not significantly associated with counseling. Regarding treatment adherence, X1, X2, X3, X4, X5, and X6 demonstrated significant direct associations. X7 and X8 were not significantly directly associated with adherence. Culturally grounded counseling (X9) demonstrated a significant positive association with treatment adherence (β = 0.245, *p* = 0.003).

### 3.4. Mediation Analysis

Table 5 presents bootstrapped indirect effects. Full statistical mediation was identified for economic conditions (X7): the direct path to adherence was non-significant (β = −0.082, *p* = 0.314) while the indirect path through counseling was significant (indirect β = 0.057, *p* = 0.023). Partial statistical mediation was observed for psychological determinants (X1), cultural values and lifestyle (X4), religious and philosophical beliefs (X5), and social and kinship relationships (X6), where both direct and indirect paths were significant. No significant mediation was observed for X2, X3, or X8.

**Figure 1:**
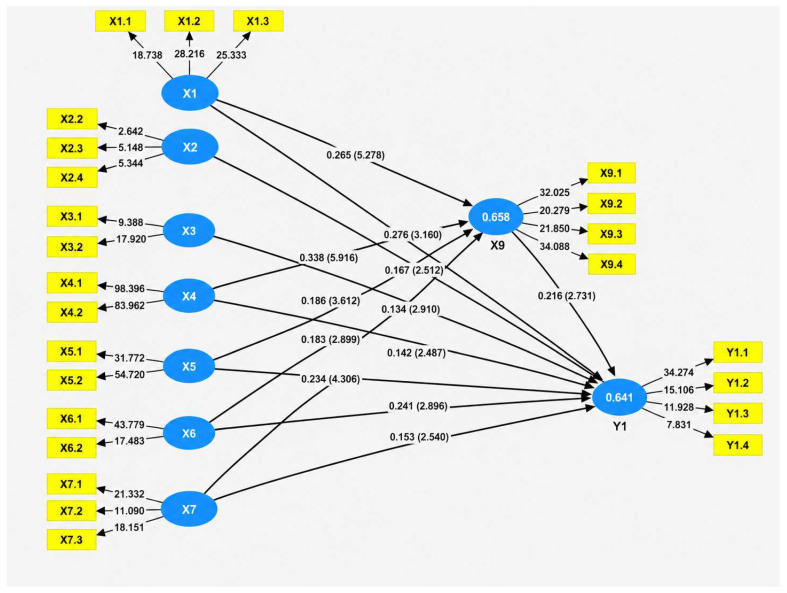
Tested structural model integrating transcultural nursing (Sunrise Model) dimensions and behavioral (IMB framework) determinants of tuberculosis treatment adherence. Note: Numbers on paths represent standardized β coefficients; R^2^ values are shown within endogenous constructs. Significant paths (*p* < 0.05) are shown as solid arrows; non-significant paths are dashed.

Figure 1 illustrates the tested structural model with standardized path coefficients.

## 4. Discussion

This study developed and tested an integrated model of TB treatment adherence by combining Leininger’s Culture Care Theory (Sunrise Model) with the IMB framework in the multi-ethnic, predominantly Christian setting of Kupang City, East Nusa Tenggara, Indonesia. The model demonstrated strong explanatory performance (R^2^ = 0.649 for adherence), supporting the view that adherence is a culturally embedded behavior shaped by psychological readiness, meaning-making, and social context, consistent with the qualitative TB adherence literature [6].

### 4.1. Measurement Quality

The measurement model demonstrated acceptable reliability and validity. ρA values (range: 0.791–0.957) confirmed reliability by the more conservative Dijkstra–Henseler criterion. Common method bias was assessed using Harman’s single-factor test (28.3% first-factor variance, below the 50% threshold); while this test has been critiqued as insufficiently sensitive when used alone [22], the supplementary VIF examination of indicator pairs (all < 3.3) provided convergent evidence that CMB is unlikely to be a dominant confound. The Durbin–Watson statistic (1.97) confirmed residual independence, and visual inspection of residual plots confirmed homoscedasticity, collectively supporting the validity of the structural model interpretation. Two removed indicators (X2.1 and X8.1) had negative loadings attributable to ceiling effects in a highly educated sample (X2.1) and a heterogeneous interpretation of a government trust item (X8.1). Two retained indicators with loadings below 0.70 (X2.2 = 0.575; Y1.4 = 0.551) were retained for theoretical reasons, with overall construct reliability and AVE remaining acceptable. The near-threshold X9–Y1 HTMT (0.832) reflects the theoretically expected proximity of counseling and adherence as proximal constructs, not construct redundancy.

### 4.2. Culturally Grounded Counseling as a Mediating Mechanism

The significant statistical association between culturally grounded counseling and treatment adherence (β = 0.245) and the mediation patterns observed are consistent with the IMB framework’s proposition that sustained health behavior depends on motivation and behavioral skills built through counseling [13]. The full statistical mediation pattern for economic conditions (X7) is particularly noteworthy: the non-significant direct association with adherence, combined with a significant indirect association with counseling, suggests that financial constraints may be associated with adherence principally through their influence on counseling engagement and content. This has direct implications for nursing practice: nurses conducting TB counseling should explicitly assess patients’ financial constraints and provide problem-solving support—by, for example, identifying transport subsidies, community food assistance programs, or flexible appointment scheduling—rather than defaulting to information-provision-only approaches [15]. Nurses are uniquely positioned to deliver this resource-linking, barrier-focused counseling within routine Puskesmas TB care contacts without requiring additional specialist referral. The partial mediation patterns for X1, X4, X5, and X6 indicate that these constructs have direct adherence associations beyond what counseling accounts for, implying that some cultural, psychological, and social dimensions operate through mechanisms independent of the counseling encounter.

### 4.3. Cultural, Psychological, and Social Determinants in the Kupang Context

Cultural values and lifestyle (X4) demonstrated the strongest association with counseling engagement (β = 0.345, f^2^ = 0.272), reflecting the prominent role of adat obligations and communal ceremonies in NTT that can create competing demands on daily medication schedules. Religious and philosophical beliefs (X5) showed the largest direct effect on adherence (β = 0.268, f^2^ = 0.125): in predominantly Christian NTT communities, faith networks can function as stigma-reduction channels and treatment encouragement sources, making this dimension particularly salient for counseling. Social and kinship relationships (X6) reflect the extended clan (suku) structures of Timorese, Rotenese, and Savunese societies, where collective decision-making about health and caregiver accompaniment operate within kinship networks.

The strong role of psychological determinants (X1) in predicting both counseling engagement and adherence is consistent with the IMB model’s proposition that psychological readiness is a prerequisite for behavioral skill development [13]. Education (X2) and technology (X3) showed significant direct associations with adherence but not with counseling, suggesting that these factors support adherence through comprehension, regimen management, and digital reminders, mechanisms that do not require culturally aligned counseling as an intermediary [24]. This is consistent with recent evidence demonstrating that digital health interventions (e.g., SMS medication reminders, video-observed therapy) can improve TB medication adherence independent of cultural counseling content, though their acceptability and effectiveness may be moderated by stigma and social context [24,25]. The non-significant associations for political–legal context (X8) may reflect limited score variability following indicator removal, rather than genuine irrelevance of policy environments in TB control.

### 4.4. Comparison with Prior Models

The explanatory power of the proposed model (R^2^ = 0.649 for adherence) compares favorably with comparable SEM-based TB adherence models in LMIC settings, which typically explain 30–50% of adherence variance when using single-domain determinants. The relative strength of cultural values and lifestyle as the strongest predictor of counseling engagement diverges from studies conducted in Javanese settings, where knowledge and family support have been more consistently identified as primary drivers [15]. It should be noted that the study by Widjanarko et al. [15], the primary Indonesian reference for social/kinship relationships, was conducted in the Javanese cultural context, which differs substantially from the clan-based kinship systems of NTT. Nevertheless, the underlying principle—that kinship networks moderate adherence through practical and normative support—is consistent across both settings. This divergence supports the premise that adherence models require context-specific validation.

### 4.5. Alternative Explanations and Limitations

Several alternative explanations and potential confounds warrant consideration. First, reverse causation cannot be excluded: patients who adhere more consistently may also engage more actively with counseling sessions. Second, unmeasured variables—including disease severity, number of counseling contacts, nurse communication quality, and household-level dynamics—may confound observed associations. Third, since counseling receipt and adherence were both self-reported in the same questionnaire session, inflated correlations due to common method variance cannot be entirely excluded, although the Harman test result (28.3%) suggests that this is unlikely to be dominant. Fourth, cultural response styles may partly explain observed associations between cultural constructs and adherence.

These limitations are inherent to the cross-sectional descriptive design: the absence of longitudinal follow-up means that temporal precedence—a requirement for causal inference—cannot be established and that changes in adherence behavior over the treatment course remain unexamined. Furthermore, the single-site, single-region sample limits the generalizability of both the structural coefficients and the instrument’s psychometric properties to populations with substantially different cultural, religious, or socioeconomic profiles. Regarding applicability beyond Kupang, Indonesia’s vast cultural, linguistic, and religious diversity means that specific counseling content and item wording cannot be directly transferred without systematic cultural adaptation and re-validation. Counseling strategies appropriate in the Christian-majority, adat-influenced communities of NTT may differ markedly from those needed in Muslim-majority Java, Aceh, or West Sumatra or in indigenous communities of Kalimantan and Papua. The integrated model’s theoretical architecture—positioning culturally grounded counseling as a mediating mechanism—is proposed as a generalizable structural framework, but its indicator items require context-specific adaptation. Multi-site replication across diverse Indonesian provinces is a research priority.

## 5. Conclusions

Tuberculosis treatment adherence is most fully understood as a culturally embedded behavioral process shaped by the intersection of psychological readiness, cultural belief systems, social relationships, and structural conditions. This study developed and validated a theory-informed structural model integrating Leininger’s Transcultural Nursing Theory (Sunrise Model) and the IMB framework, providing cross-sectional evidence that culturally grounded counseling is statistically associated with TB treatment adherence and statistically mediates relationships between five antecedent determinants and adherence (full mediation for economic conditions; partial mediation for psychological, cultural, religious, and social determinants) in the multi-ethnic, predominantly Christian setting of Kupang City, East Nusa Tenggara (R^2^ = 0.658 for counseling; R^2^ = 0.649 for adherence). Causal conclusions cannot be drawn from these cross-sectional data, and the model requires prospective longitudinal and interventional validation before clinical implementation can be recommended. The integrated framework and validated instrument offer a replicable foundation for future multi-site studies and culturally adapted counseling intervention development in TB care.

## Figures and Tables

**Table 1:** Participant Characteristics (n = 172).

Characteristic	Category	n	%
Age (years)	19–44	118	68.6
	45–59	36	20.9
	≥60	18	10.5
Sex	Male	94	54.7
	Female	78	45.3
Education level	No formal education	3	1.7
	Primary school	23	13.4
	Junior high school	12	7.0
	Senior high school	98	57.0
	Higher education	36	20.9
Occupation	Civil servant	7	4.1
	Military/Police	1	0.6
	Retired	3	1.7
	Private employee	19	11.0
	Self-employed	40	23.3
	Homemaker	30	17.4
	Unemployed	27	15.7
	Other	45	26.2
Treatment phase	Intensive (months 1–2: 2HRZE)	65	37.8
	Continuation (months 3–6: 4HR)	107	62.2

**Table 2:** Measurement Model Properties. CR = Composite Reliability; ρA = Dijkstra–Henseler’s rho_A; AVE = Average Variance Extracted. † Retained despite loading < 0.70 due to theoretical irreplaceability and acceptable construct-level fit.

Construct	Item	Loading	CR	ρA	AVE	Note
X1—Psychological Determinants	X1.1	0.772	0.860	0.791	0.672	
	X1.2	0.855				
	X1.3	0.831				
X2—Educational Factors	X2.2	0.575 †	0.853	0.826	0.669	
	X2.3	0.872				
	X2.4	0.958				
X3—Technological Factors	X3.1	0.808	0.888	0.852	0.801	
	X3.2	0.974				
X4—Cultural Values & Lifestyle	X4.1	0.963	0.959	0.957	0.921	
	X4.2	0.957				
X5—Religious & Philosophical Beliefs	X5.1	0.867	0.886	0.851	0.795	
	X5.2	0.916				
X6—Social & Kinship Relationships	X6.1	0.912	0.870	0.836	0.771	
	X6.2	0.842				
X7—Economic Conditions	X7.1	0.870	0.845	0.808	0.645	
	X7.2	0.763				
	X7.3	0.772				
X8—Political & Legal Context	X8.2	0.913	0.896	0.857	0.812	
	X8.3	0.889				
X9—Culturally Grounded Counseling	X9.1	0.825	0.885	0.847	0.657	
	X9.2	0.793				
	X9.3	0.803				
	X9.4	0.821				
Y1—TB Treatment Adherence	Y1.1	0.885	0.842	0.791	0.577	
	Y1.2	0.818				
	Y1.3	0.744				
	Y1.4	0.551 †				
Removed: X2.1	—	−0.077	—	—	—	Below threshold
Removed: X8.1	—	−0.317	—	—	—	Below threshold

**Table 3:** HTMT Discriminant Validity Matrix. * Near-threshold value (0.832 < 0.85); reflects theoretical proximity of X9 and Y1 as proximal constructs.

	X1	X2	X3	X4	X5	X6	X7	X8	X9	Y1
X1	—									
X2	0.132	—								
X3	0.291	0.055	—							
X4	0.438	0.156	0.217	—						
X5	0.515	0.102	0.212	0.400	—					
X6	0.408	0.199	0.162	0.147	0.467	—				
X7	0.388	0.163	0.152	0.148	0.579	0.731	—			
X8	0.309	0.055	0.101	0.260	0.481	0.218	0.288	—		
X9	0.735	0.106	0.211	0.625	0.706	0.634	0.679	0.333	—	
Y1	0.782	0.229	0.371	0.610	0.751	0.568	0.440	0.288	0.832 *	—

**Table 4:** Structural Model Results. β = standardized path coefficient; t = bootstrapped t-statistic (5000 resamples); f^2^ effect sizes: small ≥ 0.02, medium ≥ 0.15, large ≥ 0.35.

Path	β	t	*p*	f^2^	Effect Size	Decision
X1 → X9	0.274	5.309	<0.001	0.160	Medium	Supported
X1 → Y1	0.227	3.149	0.002	0.092	Small–medium	Supported
X2 → X9	0.008	0.169	0.866	0.000	—	Not supported
X2 → Y1	0.149	2.233	0.026	0.059	Small	Supported
X3 → X9	−0.034	0.705	0.481	0.003	—	Not supported
X3 → Y1	0.131	1.969	0.049	0.043	Small	Supported
X4 → X9	0.345	5.547	<0.001	0.272	Medium–large	Supported
X4 → Y1	0.135	2.210	0.027	0.032	Small	Supported
X5 → X9	0.195	3.434	0.001	0.073	Small	Supported
X5 → Y1	0.268	3.027	0.003	0.125	Small–medium	Supported
X6 → X9	0.185	2.883	0.004	0.068	Small	Supported
X6 → Y1	0.181	3.004	0.003	0.060	Small	Supported
X7 → X9	0.232	4.236	<0.001	0.102	Small–medium	Supported
X7 → Y1	−0.082	1.008	0.314	0.011	—	Not supported
X8 → X9	−0.025	0.506	0.613	0.002	—	Not supported
X8 → Y1	−0.053	0.989	0.323	0.007	—	Not supported
X9 → Y1	0.245	2.937	0.003	0.058	Small	Supported

**Table 5:** Statistical Mediation Effects of Culturally Grounded Counseling (X9). Full mediation = non-significant direct path + significant indirect path. Partial mediation = both significant.

Path	Indirect β	t	*p*	Mediation Type
X1 → X9 → Y1	0.067	2.396	0.017	Partial mediation (direct β = 0.227, *p* = 0.002)
X2 → X9 → Y1	0.002	0.168	0.867	No mediation
X3 → X9 → Y1	−0.008	0.675	0.500	No mediation
X4 → X9 → Y1	0.084	2.904	0.004	Partial mediation (direct β = 0.135, *p* = 0.027)
X5 → X9 → Y1	0.048	2.150	0.032	Partial mediation (direct β = 0.268, *p* = 0.003)
X6 → X9 → Y1	0.045	1.989	0.047	Partial mediation (direct β = 0.181, *p* = 0.003)
X7 → X9 → Y1	0.057	2.282	0.023	Full mediation (direct β = −0.082, *p* = 0.314)
X8 → X9 → Y1	−0.006	0.483	0.629	No mediation

## Data Availability

The data presented in this study are available upon reasonable request from the corresponding author, subject to ethical restrictions.

## Public Involvement Statement

No public involvement in any aspect of this research.

## Guidelines and Standards Statement

This manuscript was drafted against the STROBE (Strengthening the Reporting of Observational studies in Epidemiology) guidelines for cross-sectional research.

## Abbreviations

The following abbreviations are used in this manuscript:

AVE	Average Variance Extracted
CMB	Common Method Bias
CR	Composite Reliability
DOTS	Directly Observed Therapy Short-Course
HTMT	Heterotrait–Monotrait Ratio
IMB	Information–Motivation–Behavioral Skills
MDR/XDR-TB	Multidrug-Resistant/Extensively Drug-Resistant Tuberculosis
NTT	East Nusa Tenggara (Nusa Tenggara Timur)
Puskesmas	Primary Healthcare Center (Indonesia)
ρA	Dijkstra–Henseler’s rho_A
SEM-PLS	Structural Equation Modeling–Partial Least Squares
SRMR	Standardized Root Mean Square Residual
TB	Tuberculosis
VIF	Variance Inflation Factor
WHO	World Health Organization
